# Poisoning by Gelsemium

**Published:** 1876-11

**Authors:** 


Poisoning by Gelsemium.—The *Cincinnati Lancet and Observer* publishes a case of poisoning by gelsemium, and its successful treatment, reported by George S. Courtright, M.D., of Lithipolis, Ohio.

Poisoning by this remedy seems not to be very frequently met with, and no special antidote for such cases is generally known. It is therefore necessary, in order to afford rational treatment, that the practitioner should know what organ is affected by the poison, and what kind of action is exerted by it. Knowing this, and the physiological action of remedies generally, there is much less uncertainty in the treatment than when reliance is had solely on published specific antidotes.

The reporter truly says: "But every case should demand our care, and every known remedy should be tried, as well as those whose physiological action would seem to be indicated.

before giving up the case." Gelsemium is a nervous sedative, as the symptoms of overdose given in this case prove. They are thus described: "Head falling forward, the chin resting on the breast; respiration slow; pulse 98 to 100, and very weak; face congested; lips of a livid color; the mouth was partially open, and the lower jaw hanging as an almost useless appendage; could move the tongue slightly, but unable to articulate distinctly; interior of the mouth and fauces moist; pupils largely dilated and insensible to light; eyes had a fixed stare; sclerotic congested; lids drooping, so that it was necessary to pull them apart to see the eye; intellect seemed clear." Without any information as to the nature of the poison taken, the treatment was based on the symptoms present and the known action of remedies. The dilated pupil and other evidences of a want of nervous power, suggested the necessity for a powerful excitant of the great nervous centres, and that article which is known to contract the pupil. Accordingly, morphia, in the dose of half a grain, was injected under the skin, and repeated in a few minutes, with marked relief, followed by complete recovery.

